# Effect of prostate gland weight on the surgical and oncological outcomes of extraperitoneal robot-assisted radical prostatectomy

**DOI:** 10.1186/s12894-018-0434-4

**Published:** 2019-01-03

**Authors:** Min Seok Kim, Won Sik Jang, Doo Yong Chung, Dong Hoon Koh, Jong Soo Lee, Hyeok Jun Goh, Young Deuk Choi

**Affiliations:** 1 Department of Urology and Urological Science Institute, Yonsei University College of Medicine, 50-1, Yonsei-ro, Seodaemun-gu, Seoul, 03722 Republic of Korea; 2 Department of Urology, Severance Hospital, Urological Science Institute, Yonsei University College of Medicine, 50-1, Yonsei-ro, Seodaemun-gu, Seoul, 03722 Republic of Korea

**Keywords:** Radical prostatectomy, Prostate weight, Robot, Prostate cancer

## Abstract

**Background:**

Robot-assisted radical prostatectomy (RARP) is performed by urologists as one of the surgical procedures for treating prostate cancer. Numerous studies have been published with regard to the impact of prostate weight on performing RARP but were limited by the insufficient number of patients and use of the transperitoneal approach. This study aimed to determine the effect of prostate gland weight on the surgical and short-term oncological outcomes of RARP using the extraperitoneal approach.

**Methods:**

In total, 1168 patients who underwent extraperitoneal RARP (EP-RARP) performed by a single surgeon at Yonsei University Severance Hospital between May 2009 and May 2016 were included in the study. The patients were divided into 4 groups according to the prostate weight measured by transrectal ultrasonography preoperatively. Intraoperative and postoperative outcomes were analyzed retrospectively. One-way analysis of variance and the chi-square test were used in the statistical analyses.

**Results:**

Age, the Gleason score, clinical stage, and pathological stage were significantly different. Patients with a larger prostate size had a longer console time and higher estimated blood loss (*P* < 0.05). There were no significant differences between the 4 groups in length of hospital stay, duration of catheterization, blood transfusion, body mass index, prostate-specific antigen (PSA) level, history of abdominal surgery, intraoperative complications, positive surgical margin, incidence of lymphocele, and PSA recurrence after 1 year.

**Conclusions:**

The console time and estimated blood loss were significantly increased with a larger prostate size. However, there were no significant differences in the oncologic outcome and intraoperative complications, suggesting that EP-RARP requires meticulous bleeding control in patients with a prostate weighing > 75 g, and if appropriate management is implemented for blood loss intraoperatively, EP-RARP can be performed regardless of the prostate size.

## Background

Since Blinder and Kramer first performed robot-assisted radical prostatectomy (RARP) in 2000, RARP has been performed by numerous urologists as one of the surgical procedures for treating prostate cancer (PCa) [1]. Several studies comparing the operative outcomes of RARP with those of open radical prostatectomy (ORP) have been published and showed no significant differences in oncologic and functional outcomes, and better outcomes for bleeding in RARP [2–4].

Among the several factors affecting radical prostatectomy (RP), prostate weight has been reported by many studies. According to the studies on the impact of prostate weight on the operative outcomes of ORP, the larger the prostate size, the greater the risk of blood loss and blood transfusion, and the smaller the prostate size, the greater is the incidence of a positive surgical margin [5]. In laparoscopic RP, similar to what is observed in ORP, there is proportionate increase in the amount of bleeding as the prostate enlarges in size, and the smaller the prostate size, the higher the incidence of a positive margin [6]. Numerous studies have been published on the impact of prostate weight on RARP. However, there were limitations to these studies in that the number of patients was insufficient and most surgical methods were performed using the transperitoneal approach [7–12]. Generally, the operative space is narrower in the extraperitoneal approach than in the transperitoneal approach. Thus, it may be more difficult to perform RP using the extraperitoneal approach than to perform RP using the transperitoneal approach in the case of a larger prostate size.

Consequently, we aimed to determine whether the prostate size affects perioperative or oncological outcomes when the extraperitoneal approach is used. We retrospectively analyzed 1168 patients who underwent RARP using the extraperitoneal approach performed by a single surgeon. In this paper, we evaluated the effect of prostate gland weight on the surgical and short-term oncological outcomes of RARP performed using the extraperitoneal approach.

## Methods

After institutional review board approval (approval number: YUHS 4–2018-0555) was obtained, we retrospectively analyzed 1509 patients with PCa who underwent extraperitoneal RARP (EP-RARP) performed by a single surgeon in Yonsei University Severance Hospital between May 2009 and May 2016. In total, 1168 patients were included after excluding patients with inadequate medical records and suspected metastasis (clinical T4 or M1) on preoperative magnetic resonance imaging (MRI) of the prostate or whole body bone scan prior to surgery for prostate cancer. The operator was an expert surgeon who had previously performed 250 cases of RARP [13]. The patients were divided into 4 groups (group 1: < 25 g, group 2: 25–50 g, group 3: 51–75 g, and group 4: > 75 g) according to the prostate weight measured by transrectal ultrasonography preoperatively. The prostate weight was calculated using the following formula: height **×** length **×** width × π/6. The clinical stage was classified according to TNM staging, which was established by the American Joint Committee on Cancer in 2010 using preoperative pelvic magnetic resonance imaging and whole-body bone scanning [14]. Transfusion records were included in all cases intraoperatively and during hospitalization. Prostate-specific antigen (PSA) recurrence was defined as a continuous increase in the PSA level of 0.2 or more after 1 year [15].

## Operative technique

All RPs were performed using the extraperitoneal approach with the da Vinci Surgical System (Intuitive Surgical, Sunnyvale, CA, USA). The operation was performed using the same procedure as previously reported [16]. Six ports (4 robot arm ports and 2 assistant ports) were used for RARP. A 1.5-cm vertical infraumbilical incision was made, exposing the anterior rectus sheath. The anterior rectus sheath was incised, and the rectus abdominis muscle was swept. Then, an extraperitoneal space was created using blunt finger dissection. A PDB balloon dilator (Tyco, Princeton, NJ, USA) was used to expand the extraperitoneal cavity. A 5-mm suction port was inserted into the left upper side of the umbilicus using the left index finger. A 12-mm camera port was inserted into the infraumbilical incision site, and an 8-mm port was placed 8 cm away from the umbilicus in both directions. A 12-mm assistant port was inserted 2 cm above the left anterior superior iliac spine, and an 8-mm port was inserted 2 cm above the right anterior superior iliac spine. The endopelvic fascia was dissected. The prostatic-vesical junction was identified, and the proximal urethra was exposed. The vas deferens and seminal vesicles were separated from Denonvilliers’ fascia. The dorsal vein complex was resected without ligation, and the distal urethra was exposed. The prostate apex was separated from the apical urethra and Denonvilliers’ fascia. Both vascular pedicles were resected, and the prostate was completely dissected with the prostate specimen placed inside the entrapment bag (Lap-bag, Sejong Med, Paju, Korea). Vesicourethral anastomosis was performed using Monosyn 3–0 double arm sutures (Aesculap, Center Valley, PA, USA). In a posterior direction from the bladder, a continuous suture was started 3 times on the right side and 3 times on the left side. Then, an 18-French Foley catheter was inserted into the bladder. Suturing was performed twice on both sides. After approximating the puboprostatic collar and bladder, a knot was made.

## Histopathological analysis of the specimens

All prostate specimens were processed in accordance with a well-established protocol and reviewed by a pathologist [17]. The pathologist recorded the tumor location, extracapsular extension (ECE), seminal vesicle invasion (SVI), vas invasion, pathological Gleason score, pathological stage, and positive surgical margin. A positive surgical margin was defined as a cancer gland reaching the inked margin.

## Statistical analysis

Statistical analysis was performed using SPSS 23 software (IBM Corp., Armonk, NY, USA). One-way analysis of variance was used for normally distributed continuous data. The chi-square test was used for categorical data. Parameters with a *P*-value < 0.05 were considered statistically significant.

## Results

Of the 1168 patients, 157 were in group 1, 824 in group 2, 149 in group 3, and 38 in group 4. Patient characteristics are shown in Table 1. The larger the prostate, the older the patient’s age (*P* < 0.001). There were no significant differences in history of abdominal surgery, body mass index, and PSA level in terms of prostate weight. The larger the prostate, the lower the Gleason score (*P* < 0.05) and higher the incidence of clinical T1 stage cancer (*P* < 0.05).

**Table 1:** Patient characteristics classified according to the prostate weight

Prostate weight measured by TRUS (g)	< 25 g	25–50 g	51–75 g	> 75 g	*P*-value
Patients (n)	157	824	149	38	
Mean age (years)	64.1 ± 8.7	64.6 ± 7.4	65.9 ± 5.8	70.2 ± 5.4	< 0.001
Mean BMI (kg/m^2^)	23.8 ± 2.9	24.2 ± 2.6	25.1 ± 8.4	25.1 ± 3.4	0.07
Mean PSA level (ng/ml)	10.1 ± 9.5	10.5 ± 8.7	10.9 ± 8.6	15.9 ± 12.6	0.08
Prior abdominal surgery	29 (18.5%)	109 (13.2%)	26 (17.4%)	6 (15.8%)	0.55
Mean prostate weight by TRUS (g)	16.8 ± 2.9	31.0 ± 6.7	53.6 ± 6.7	92.1 ± 23.9	< 0.001
Mean pathologic prostate weight (g)	22.4 ± 5.2	35.4 ± 9.4	55.7 ± 10.1	81.2 ± 24.2	< 0.001
Preoperative Gleason score					0.03
2–6	57 (36.3%)	332 (40.3%)	80 (53.7%)	16 (42.1%)	
7	62 (39.5%)	274 (33.3%)	43 (28.9%)	11 (28.9%)	
8–10	38 (24.2%)	218 (26.5%)	26 (17.4%)	11 (28.9%)	
Clinical stage					0.02
T1	35 (22.3%)	152 (18.4%)	43 (28.9%)	14 (36.8%)	
T2	77 (49.0%)	430 (52.2%)	74 (49.7%)	14 (36.8%)	
T3	45 (28.7%)	242 (29.4%)	32 (21.5%)	10 (26.3%)	

*TRUS* transrectal ultrasonography, *BMI* body mass index, *PSA* prostate-specific antigen

Perioperative characteristics are presented in Table 2. Length of hospital stay and duration of catheterization were not correlated with prostate weight, and the incidence of lymphocele was not significantly correlated with prostate weight. The mean console time and mean estimated blood loss were significantly increased with increasing prostate weight (*P* < 0.001). Blood transfusion, rectal injury, lymph node dissection, and nerve sparing were not associated with prostate weight. There were no other complications, except for rectal injury intraoperatively, and there were no open conversions.

**Table 2:** Perioperative characteristics of patients classified according to the prostate weight

Prostate weight measured by TRUS (g)	< 25 g	25–50 g	51–75 g	> 75 g	*P*-value
Patients (n)	157	824	149	38	
Length of hospital stay (days)	5.5 ± 2.0	5.3 ± 2.0	5.1 ± 1.8	5.8 ± 2.9	0.30
Duration of catheterization (days)	11.0 ± 1.9	10.9 ± 2.0	11.1 ± 2.1	10.9 ± 1.8	0.49
Mean console time (min)	45.1 ± 14.8	47.0 ± 13.8	49.5 ± 14.1	61.7 ± 18.3	< 0.001
Mean estimated blood loss (ml)	347 ± 250	386 ± 262	456 ± 303	646 ± 423	< 0.001
Transfusion (n)	2 (1.3%)	2 (0.2%)	1 (0.7%)	1 (2.6%)	0.67
Rectal injury	1 (0.6%)	0 (0.0%)	2 (1.3%)	0 (0.0%)	0.44
Incidence of lymphocele	5 (3.2%)	31 (3.8%)	4 (2.7%)	2 (5.3%)	0.89
Lymph node dissection					0.88
No	115 (73.2%)	597 (72.5%)	114 (76.5%)	26 (68.4%)	
Yes	42 (26.8%)	227 (27.5%)	35 (23.5%)	12 (31.6%)	
Nerve sparing					0.89
None	8 (5.1%)	38 (4.6%)	5 (3.4%)	3 (7.9%)	
Unilateral	2 (11.1%)	14 (1.7%)	2 (1.3%)	0 (0.0%)	
Bilateral	147 (93.6%)	772 (93.7%)	142 (95.3%)	35 (92.1%)	

*TRUS*, transrectal ultrasonography

Table 3 shows the pathologic outcomes. SVI and a positive surgical margin were not significantly associated with prostate weight. There was no significant difference in PSA recurrence after 1 year in terms of prostate weight. ECE significantly increased as the prostate weight decreased (*P* < 0.05), and in the pathologic stage, T2 increased as the prostate weight increased (*P* < 0.001).

**Table 3:** Pathologic outcomes of patients classified according to the prostate weight

Prostate weight measured by TRUS (g)	< 25 g	25–50 g	51–75 g	> 75 g	*P*-value
Patients (n)	157	824	149	38	
Extracapsular extension					0.003
Negative	87 (55.4%)	430 (52.2%)	104 (69.8%)	26 (68.4%)	
Positive	70 (44.6%)	394 (47.8%)	45 (30.2%)	12 (31.6%)	
Seminal vesicle invasion					0.88
Negative	146 (93.0%)	742 (90.0%)	143 (96.0%)	32 (84.2%)	
Positive	11 (7.0%)	82 (10.0%)	6 (4.0%)	105 (9.0%)	
Pathological Gleason score					0.01
3–6	35 (22.3%)	201 (24.4%)	62 (41.6%)	12 (31.6%)	
7	86 (54.8%)	419 (50.8%)	57 (38.3%)	17 (44.7%)	
8–10	36 (22.9%)	204 (24.8%)	30 (20.1%)	9 (23.7%)	
Pathological stage					0.01
T2	85 (54.1%)	434 (52.7%)	103 (69.1%)	25 (65.8%)	
T3	72 (45.9%)	379 (46.0%)	46 (30.9%)	12 (31.6%)	
T4	0 (0.0%)	11 (1.3%)	0 (0.0%)	1 (2.6%)	
Positive surgical margin	60 (38.2%)	351 (42.6%)	51 (34.2%)	10 (26.3%)	0.12
PSA recurrence (1 year)	16 (10.2%)	127 (15.4%)	14 (9.4%)	4 (10.5%)	0.65

*TRUS* transrectal ultrasonography

## Discussion

This study’s results showed that EP-RARP is as feasible as transperitoneal RARP (TP-RARP) regardless of the prostate weight. In our study, the greater the prostate weight, the lower the Gleason score and lower the T stage. This result is thought to be the effect of a lead time bias, as described by Link et al. [7] Benign prostatic hyperplasia (BPH) can lead to an increased PSA level, so patients with BPH are more likely to be diagnosed at a relatively early stage; in addition, prostate biopsy is performed more often in patients with BPH than in those without BPH [18]. In our study, ECE significantly increased in patients with a lower prostate weight, and the T stage was increased more in patients with a low prostate weight than in those with a high prostate weight. Our results are consistent with those of previous studies. Briganti et al. [19] reported that PCa in smaller glands is more aggressive and therefore there are higher rates of ECE than when PCa involves larger glands. Hong et al. [20] reported that prostate size is not useful in predicting tumor recurrence, but it is associated with the progression of PCa. These explanations have not yet been clearly elucidated. However, several hypotheses have been developed, including the following. First, men with a small prostate secrete less testosterone. Low testosterone levels are associated with progressive PCa [21, 22]. Second, the benign tissues of BPH play an inhibitory role in cancer cell progression [21, 23].

In our study, there was no correlation between the prostate size and a positive surgical margin in RARP. However, Allaparthi et al. [8] and Link et al. [7] reported that the lower the prostate weight, the more positive the surgical margin is. This is because a small-sized prostate has a higher cancer density than a large-sized prostate [24].

The mean console time and mean estimated blood loss were significantly increased with increasing prostate weight in our study. Boylu et al. [9] and Yasui et al. [10] reported that the greater the prostate weight, the longer the operative time, and Hirasawa et al. [11] reported that the greater the prostate weight, the more the blood loss, as it is thought that men with enlarged prostate glands usually have more vascularity and broader resection margins. However, the transfusion rate was not significantly associated with the prostate weight, so even though there is a greater amount of bleeding with a greater prostate weight, it would not be clinically meaningful. Surgeons feel technically challenged when operating on an enlarged prostate because BPH affects the structure of the prostate in terms of its size and shape [25]. As mentioned previously, the large amount of bleeding and broad resection margin may have prolonged the console time.

Numerous studies have been published about the impact of the prostate weight on performing RARP, but the surgical technique most commonly used was the transperitoneal approach. Most studies reported that using the transperitoneal approach was feasible regardless of the prostate weight [7–12].

EP-RARP is associated with a lower incidence of bowel injury and postoperative hernia compared to TP-RARP, and there are no reports of symptoms of peritoneal irritation caused by gas, urinary leakage, or bleeding. However, there is a disadvantage in that the operative space is narrow and accompanied by difficulty in obtaining operative visibility [16, 26]. Because of these advantages and disadvantages, we felt that it was necessary to study the extraperitoneal approach.

According to previous studies conducted using the extraperitoneal approach, Boczko et al. [27] reported that blood loss is associated with a greater prostate weight. Allaparthi et al. [8] reported that the prostate weight is associated with a positive surgical margin. However, the study by Boczko et al. has some limitations. In particular, the statistical reliability of the data was not good, and the effect of the small prostate was not determined because the prostate weight was divided into only 2 categories [27]. The study by Allaparthi et al. was limited in terms of statistical reliability of the data because the numbers of patients with a prostate weight < 30 g and > 80 g were small [8]. In comparison, our study analyzed more patients than those of prior studies on the extraperitoneal approach and had an increased power of data analysis.

There are some limitations to our study. First, the number of patients was higher than that of prior studies, but the number of patients with a prostate weight > 70 g was relatively small compared to that in the other groups. Second, the follow-up period was relatively short (12 months), so the long-term evaluation of biochemical recurrence was not possible. Third, as all procedures were performed by a single expert surgeon, it was difficult to generalize the results. Finally, we did not perform multiple linear regression analyses of factors associated with operative outcomes. If we had performed it, we would have been able to increase the validity of the study.

## Conclusions

We found that the prostate size did not significantly affect the oncologic outcomes or surgical complications in performing EP-RARP. There was also no significant difference in the length of hospital stay and duration of catheterization. In this study, the size of the prostate was correlated with the amount of estimated blood loss and console time. However, there were no statistically significant negative operative outcomes, such as transfusion, longer hospitalization, longer catheterization, operative complications, or oncologic outcomes due to increased estimated blood loss. Therefore, we concluded that we could perform EP-RARP safely regardless of the size of the prostate.

## Abbreviations

BPH: benign prostatic hyperplasia
ECE: extracapsular extension
EP-RARP: extraperitoneal robot-assisted radical prostatectomy
ORP: open radical prostatectomy
PCa: prostate cancer
PSA: prostate-specific antigen
RARP: robot-assisted radical prostatectomy
RP: radical prostatectomy
SVI: seminal vesicle invasion
TP-RARP: transperitoneal robot-assisted radical prostatectomy
